# Evaluating chemical and surface stability of thermoformed and 3D-printed clear aligners after intraoral exposure

**DOI:** 10.1371/journal.pone.0345179

**Published:** 2026-04-02

**Authors:** Fabrizio Sanna, Leila Es Sebar, Isabella Sannino, Samuele Avolese, Simone Parrini, Marco Posadino, Paola Testa, Nicola Scotti, Mario Alovisi

**Affiliations:** 1 Department of Surgical Sciences, Dental School, Universitá di Torino, Turin, Italy; 2 Department of Mechanical and Aerospace Engineering, Politecnico di Torino, Turin, Italy; 3 Department of Applied Science and Technology, Politecnico di Torino, Turin, Italy; Ohio State University, UNITED STATES OF AMERICA

## Abstract

**Introduction:**

Align Technology, Inc. is a leading manufacturer of orthodontic aligners, using SmartTrack^TM^ (LD30), a multilayer aromatic thermoplastic polyurethane/copolyester, and the vacuum thermoforming process. Recently, direct 3D printing using Tera Harz TC-85 DAC resin has also been explored for the production of aligners. Both aligner materials are exposed to physical and chemical aging in the oral cavity. This study evaluates the chemical aging of these materials after 7 and 14 days of exposure to the oral environment, using Optical Microscopy, Raman spectroscopy, X-ray fluorescence (XRF), and X-ray diffraction (XRD).

**Materials and methods:**

Invisalign® aligners (SmartTrack^TM^ LD30) and 3D printed aligners (Tera Harz TC-85 DAC) were worn by 20 patients for 7 (*t*_1_) and 14 days (*t*_2_). The aligners were characterized initially (*t*_0_) using Optical Microscopy, Raman spectroscopy, XRF, and XRD. After *t*_1_ and *t*_2_, each aligner was reanalyzed at three locations: right and left first molars (*p*_1_ and *p*_2_), and central incisor (*p*_3_). The results were compared with never-worn aligners (*t*_0_, n = 3).

**Results:**

Both materials showed high homogeneity across all locations (*p*_1_, *p*_2_, *p*_3_) and time points, indicating uniform performance after oral exposure. PCA analysis performed on Raman spectra revealed no significant chemical changes in the surface composition after 7–14 days of use.

**Discussion and conclusions:**

The results demonstrated that exposure to the oral environment for up to 14 days did not significantly alter the surface chemistry of either aligner material. These findings are consistent with recent literature, suggesting that short-term intra-oral exposure does not affect aligner materials’ chemical stability.

## Introduction

Over recent years, clear aligner therapy (CAT) has gained significant traction as a patient-centered and aesthetically driven alternative to conventional fixed orthodontic appliances. This preference is mainly attributed to the aligners’ inherent transparency, making them nearly imperceptible during use, thereby enhancing patient comfort and aesthetic satisfaction. Consequently, CAT presents an appealing option for individuals seeking to address mild to moderate dental malocclusion without compromising their appearance [1].

Beyond aesthetics, CAT offers several clinical advantages. Studies have demonstrated improved periodontal health and reduced levels of periodontopathic bacteria compared to traditional fixed appliances, also due to the simplified oral hygiene maintenance [2]. Furthermore, CAT has shown potential for shortening treatment time in certain cases [3], and is associated with decreased soft tissue irritation, contributing to enhanced patient comfort and compliance. CAT utilizes a series of custom-designed, sequentially worn transparent aligners to apply gradual forces, effectively treating malocclusion. Aligners are typically replaced every 1–2 weeks to achieve the desired orthodontic movements [4].

The advancement of CAT has been significantly driven by innovations in transparent thermoplastic materials, coupled with computer-aided design and manufacturing (CAD-CAM) technologies and complex tooth-movement simulation software. This technology provides an aesthetic orthodontic solution that is particularly favored by adult patients.

The clear aligner market has seen significant growth, with numerous companies introducing innovative production techniques and materials. Among these, Align Technology, Inc., a global leader in thermoformed aligners, pioneered the use of transparent thermoplastic polymers with the Invisalign® system in 1998, utilizing CAD/CAM technology. Before this, clear aligners were primarily limited to minor orthodontic movements or retention following conventional fixed orthodontic therapy [5].

The Invisalign® system employs SmartTrack^TM^ (LD30), a multilayer aromatic thermoplastic polyurethane/copolyester, for its aligners. The manufacturing process involves: 3D scanning of the patient’s dentition obtained through an initial plaster impression or an intraoral scan, digital treatment planning with algorithmic segmentation with specific software that analyzes the dentination and enables precise manipulation of tooth positions, sequential tooth movement simulation, 3D printing of physical models, vacuum thermoforming of aligners and finally, trimming [6,7]. While vacuum thermoforming remains the prevalent manufacturing method for clear aligner fabrication, a rapidly emerging technology involves direct 3D printing the aligner from digital design [8]. The advent of direct 3D printing represents a significant advancement in clear aligner fabrication [9], driving the development of materials with optimized intraoral properties. Currently, Tera Harz TC-85 DAC (Graphy, Seoul, South Korea) is a CE Class IIa-certified resin approved for commercial direct 3D printing of aligners, offering an alternative to conventional thermoforming [10,11]. Eliminating intermediate models, direct 3D printing from CAD designs streamlines production, significantly reducing processing time, labor, and errors associated with analog impressions [12]. This approach, through additive manufacturing, enables precise customization of aligner dimensions to individual patient anatomy, leading to a more environmentally friendly production process with reduced waste and lower costs [13]. Reduced production steps further shorten treatment planning and manufacturing time [11], while enhancing patient accessibility.

Furthermore, direct 3D printing provides precise control over aligner properties [14], facilitating targeted pressure and reinforcement zones to improve dental movement efficiency and anchorage [15]. Regional thickness adjustments, as proposed by Grant et al. [16], enhance treatment predictability and mitigate potential side effects.

An ideal clear aligner material should exhibit excellent biocompatibility along with optimal mechanical, optical, thermal, and chemical properties. However, regardless of the manufacturing method, clear aligners could be susceptible to progressive degradation in the oral environment. Exposure to saliva, food, and beverages could induce chemical and physical alterations in the polymeric structure, compromising the mechanical properties and leading to deformation. These alterations might negatively impact orthodontic treatment efficacy, increase fracture risk, and facilitate bacterial biofilm formation, potentially impacting oral health. Therefore, understanding aligner degradation mechanisms is crucial for developing improved materials and clinical protocols. Although the clinical performance of thermoformed aligners, such as Invisalign®, is well-documented, long-term clinical data for 3D-printed aligners are limited.

Existing research on Tera Harz TC-85 DAC 3D-printed aligners primarily focuses on in vitro assessments of dimensional accuracy, thickness [10,12,17], mechanical properties [17–19], cytotoxicity [20,21], degree of conversion [17,22], and the impact of UV light during printing [17,23]. Despite these advancements, most studies have been conducted in vitro, and the effects of intraoral exposure on the properties of 3D-printed materials remain largely unexplored. Some in vivo studies have investigated thickness [24], intraoral aging effects on mechanical properties [25], and surface properties like roughness and porosity [26]. Thus, the long-term clinical behavior of 3D-printed aligners requires further investigation. However, comprehensive clinical data on the long-term performance of 3D-printed aligners are still limited. A deeper understanding of the degradation mechanisms of 3D-printed aligners is essential for developing improved materials and optimizing clinical protocols to enhance treatment efficacy. Therefore, rigorous in vitro and in vivo evaluations are necessary to ensure the safety, durability, and long-term clinical performance of these aligners. Non-destructive analytical techniques can provide valuable insights into the chemical and structural properties of aligners. Among these, Raman spectroscopy has demonstrated effectiveness in tracking molecular changes [27–29], X-ray fluorescence spectroscopy (XRF) is useful for assessing elemental composition [30,31], X-ray diffraction (XRD) can determine the crystalline structure of polymers [32], and Optical Microscopy can be employed to evaluate the surface morphology [33].

This in vivo study employs a multi-analytical approach integrating Optical Microscopy, Raman spectroscopy, XRF, and XRD to comprehensively characterize the material properties of SmartTrack^TM^ (Invisalign®) and Tera Harz TC-85 DAC aligners following oral exposure for 7 and 14 days (average daily wear: 20–22 hours). This methodology aims to provide a more detailed understanding of the effects of intraoral conditions on these materials.

## Materials and methods

This study included a sample of 20 patients recruited in a period from 1st March 2022 to 1st December 2022, with 10 undergoing orthodontic treatment with Invisalign® SmartTrack^TM^ (LD30) aligners (Align Technology, Inc., San Jose, California, USA) and 10 using direct 3D-printed aligners fabricated from Tera Harz TC-85 DAC resin (Graphy, Seoul, South Korea). The study was performed at the Department of Surgical Science of the Dental School, Università di Torino, with a 22-hour per day protocol. The study has received ethical approval from the Ethics Committee of the Città della Salute e della Scienza di Torino (no. 27/2022). Informed consent for the processing of personal data for research and scientific publication purposes has been provided by the patients in written form, and no minor patients were involved in the study.

Patients were selected based on specific inclusion criteria, which required them to be 18 years or older, have periodontal pocket depths of 4 mm or less, a plaque index below 15%, minimal or no dental mobility, absence of stabilized or persistent occlusal trauma, no carious lesions, and demonstrated motivation for orthodontic treatment.

Each patient wore one aligner for a period of 7 days (*t*_1_) and the subsequent aligner for 14 days (*t*_2_). A total of 40 aligners were analyzed, including 20 SmartTrack^TM^ aligners and 20 Tera Harz TC-85 DAC aligners. Samples of unused aligners (*t*_0_), namely n = 3 SmartTrack^TM^ and n = 3 Tera Harz TC-85 DAC, were analysed to evaluate polymer homogeneity and to set the baseline.

### Optical microscopy

Surface morphology of the aligners was examined using a Zeiss Stereo Discovery V12 microscope. Images were captured with an Axiocam 208 color camera (Carl Zeiss Microscopy GmbH, Jena, Germany) at magnifications of 20x and 40x.

Microscopic images were acquired at baseline (*t*_0_), 7 days (*t*_1_), and 14 days (*t*_2_) of intraoral exposure. The aligner surfaces of the working cusp of the first right molar (*p*_1_), the working cusp of the first left molar (*p*_2_), and the incisal edge of the central incisor (*p*_3_) were evaluated across the aforementioned magnifications, enabling a detailed visual analysis of surface changes before and after oral exposure. This approach facilitated the assessment of potential surface alterations resulting from clinical use.

### X-ray fluorescence spectroscopy (XRF)

X-ray fluorescence spectroscopy, a non-destructive analytical technique, was employed to determine the elemental composition of the aligner materials. A Bruker Tracer 5i analyzer, equipped with a 20 mm^2^ silicon drift detector and a Rhodium (Rh) anode, was employed for this analysis. Operating conditions were set as follows: accelerating voltage of 40 kV, current of 100 *μ*A, and a 3 mm collimator. XRF spectra were processed using Artax Spectra software (version 8.0.0.476).

XRF analyses were performed on the surface of the SmartTrack^TM^ and Tera Harz TC-85 DAC aligners at the baseline time point (*t*_0_), and after 7 days (*t*_1_) and 14 days (*t*_2_) of intraoral exposure.

### X-ray diffraction

X-ray diffraction (XRD) analysis was conducted to determine the crystalline structure and phase composition of the materials under investigation. A PANalytical X’Pert PRO diffractometer was operated with generator settings of 40 mA and 40 kV, and data were collected over a 2*θ* range of 10–70. Phase identification was performed using HighScore Plus software.

X-ray diffraction analyses were performed on a sample of unused aligners of SmartTrack^TM^ and Tera Harz TC-85 DAC) (*t*_0_) and also on aligners worn by patients for 7 days (*t*_1_) and 14 days (*t*_2_).

### Raman spectroscopy

Raman spectroscopy was performed using a portable BWTeK Raman spectrometer equipped with a 785 nm laser and a BAC151 Raman microscope. Non-destructive measurements were acquired directly on the surface of the aligners with a spot size of approximately ∼50 *μ*m. The emitted signal was collected by a BTC675N® spectrometer within a spectral range of 65 cm^−1^ to 3350 cm^−1^, with a resolution of 6 cm^−1^.

Raman analysis focused on the outermost layer of the material, examining three specific positions on each aligner: the working cusp of the first right molar (*p*_1_), the working cusp of the first left molar (*p*_2_), and the incisal edge of the central incisor (*p*_3_). These locations were selected to assess material behavior in areas subjected to varying levels of mechanical stress. Measurements were conducted directly on the sample surface using the following parameters: laser power of 20 mW, an integration time of 10 s, and 16 repetitions per area. This analytical approach enabled the evaluation of intraoral aging effects by comparing different surfaces of the aligners.

Raman spectra were initially acquired at *t*_0_ (unworn aligners, *t*_0_, n = 3 SmartTrack^TM^ and n = 3 Tera Harz TC-85 DAC) from the three selected points of interest (*p*_1_, *p*_2_, and *p*_3_) to assess material homogeneity before intraoral exposure. Subsequent measurements were performed at *t*_1_ (7 days) and *t*_2_ (14 days) on aligners worn intraorally to identify potential spectral changes compared to *t*_0_. Intraoral aging at the individual patient level was assessed by comparing spectra across different time points.

The acquired Raman spectra were processed using Principal Component Analysis (PCA) implemented by a Python script. Initially, only the region of interest within the spectrum was selected, focusing on the range between 1500 cm^−1^ and 1800 cm^−1^, where the most intense peaks associated with various functional groups in the polymers are located both for the SmartTrack^TM^ and Tera Harz TC-85 DAC according to literature [34,35]. Baseline correction was then applied using an asymmetric least-squares smoothing algorithm, followed by Savitzky–Golay filtering to compute the first derivative and a Standard Normal Variate (SNV) transformation prior to PCA; this preprocessing enhances subtle spectral differences and reduces the influence of residual baseline curvature, improving the sensitivity of PCA to changes in peak position and shape rather than absolute intensity.

## Results

The images acquired with the optical microscope show the working surfaces of the two types of aligners at three different time points.

**Fig 1:**
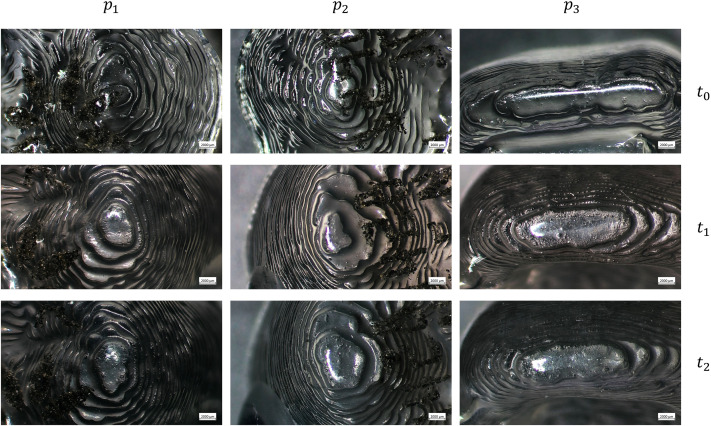
Optical microscope images of the SmartTrack^TM^ aligners at the three points examined (right first molar *p*_1_, left first molar *p*_2_, and incisal edge *p*_3_) at baseline (*t*_0_), after 7 days (*t*_1_), and after 14 days (*t*_2_) of intraoral wear, with a magnification of 20x.

**Fig 2:**
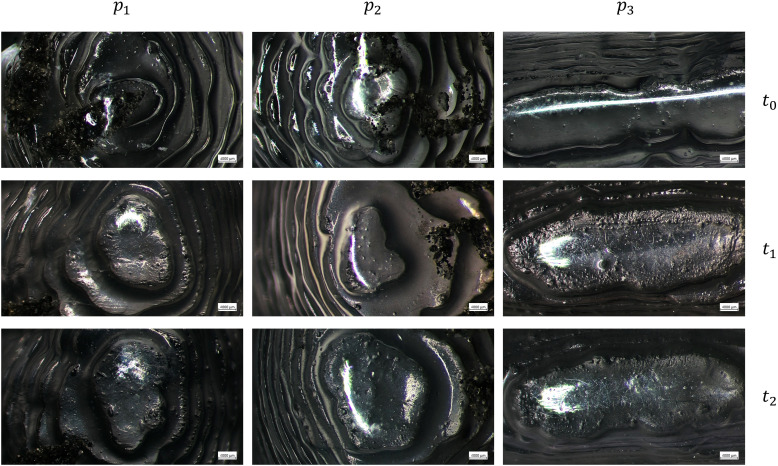
Optical microscope images of SmartTrack^TM^ aligners at the three points examined (right first molar *p*_1_, left first molar *p*_2_, and incisal edge *p*_3_) at baseline (*t*_0_), after 7 days (*t*_1_), and after 14 days (*t*_2_) of intraoral wear, with a magnification of 40x.

In particular, Fig 1 and Fig 2 show the images of SmartTrack^TM^ aligners at *t*_0_, *t*_1_, and *t*_2_, at 20x and 40x, respectively. From their comparison, signs of wear can be observed, and microfractures (micro cracks) are visible on aligner surfaces at *t*_1_, and *t*_2_, which are not present at *t*_0_. Additionally, the surface at *t*_0_ appears glossier compared to the later time points in which the surface is more opaque.

**Fig 3:**
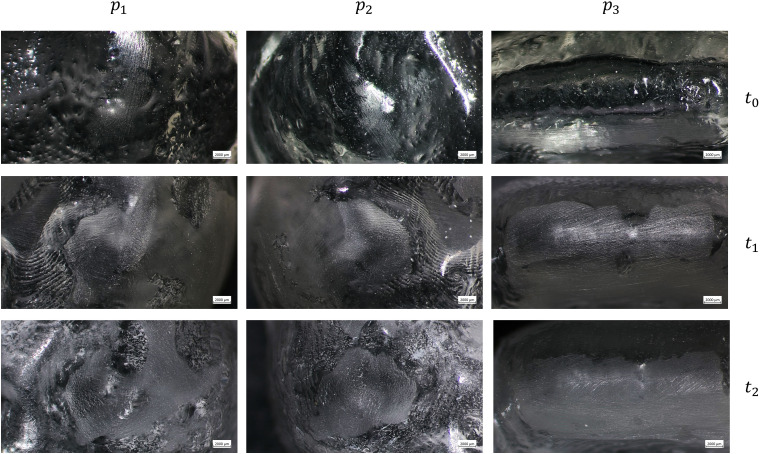
Optical microscope images of the TC-85 DAC aligners at the three points examined (right first molar *p*_1_, left first molar *p*_2_, and incisal edge *p*_3_) at baseline (*t*_0_), after 7 days (*t*_1_), and after 14 days (*t*_2_) of intraoral wear, with a magnification of 20x.

**Fig 4:**
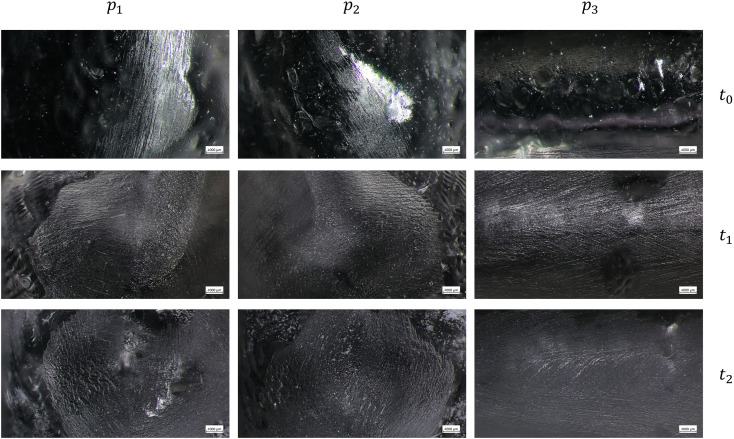
Optical microscope images of the of the TC-85 DAC aligners at the three points examined (right first molar *p*_1_, left first molar *p*_2_, and incisal edge *p*_3_) at baseline (*t*_0_), after 7 days (*t*_1_), and after 14 days (*t*_2_) of intraoral wear, with a magnification of 40x.

Fig 3, Fig 4 report the surface images of TC-85 DAC at 20x and 40x, respectively, at *t*_0_, *t*_1_, and *t*_2_. Some irregularities of the surface can be observed at *t*_0_, but they are not due to use, but rather to the manufacturing process itself, and in particular from the imprint left by the curing plate on the resin. At *t*_1_, and *t*_2_, the initial surface texture is replaced by wear areas that appear more opaque compared to *t*_0_.

**Fig 5:**
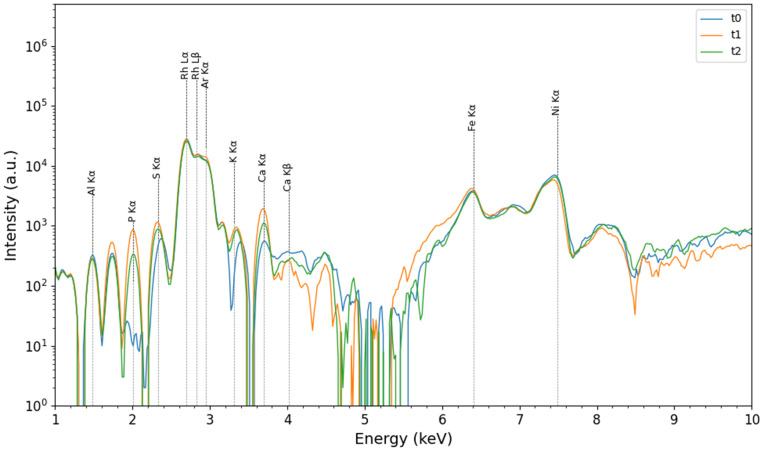
Representative XRF Spectra. XRF spectra for SmartTrack^TM^ aligner at baseline (*t*_0_), after 7 days (*t*_1_), and after 14 days (*t*_2_) of intraoral wear.

**Fig 6:**
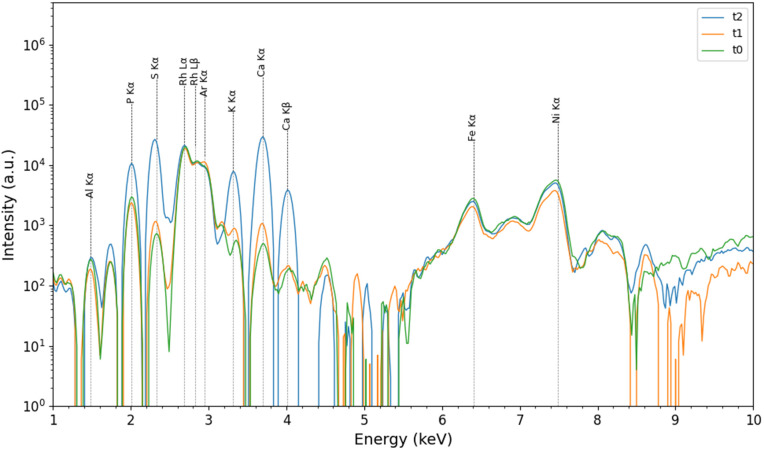
Representative XRF Spectra. XRF spectra for Tera Harz TC-85 DAC aligner at baseline (*t*_0_), after 7 days (*t*_1_), and after 14 days (*t*_2_) of intraoral wear.

XRF spectra obtained from the analysis of SmartTrack^TM^ and Tera Harz TC-85 DAC aligners at *t*_0_, *t*_1_, *t*_2_ are illustrated in Fig 5 and Fig 6, respectively.

The following elements were identified in both typology of aligners: aluminum (Al; 1.48 keV-K*α*), phosphorus (P; 2.01 keV-K*α*), sulfur (S; 2.32 keV-K*α*) rhodium (Rh; 2.69 keV-L*α* and 2.83 keV-L*β*), argon (Ar, 2.95 keV-K*α*), potassium (K; 3.31 keV-K*α*), calcium (Ca; 3.69 keV-K*α* and 4.01 keV-K*β*), iron (Fe; 6.40 keV-K*α*), and nickel (Ni; 7.48 keV-K*α*). Rh lines are attributed to the anode contribution, while the signals for Fe, Ni, and Al are considered instrumental contributions. It is worth noticing that the intensity of the lines of P, S, Ca and K increase in intensity in both materials after exposure to the oral cavity. This can be explained considering the effect of localised calcification processes of the biofilm, as reported in [34,36,37] and which may be due to the exposure to the oral environment and the saliva.

**Fig 7:**
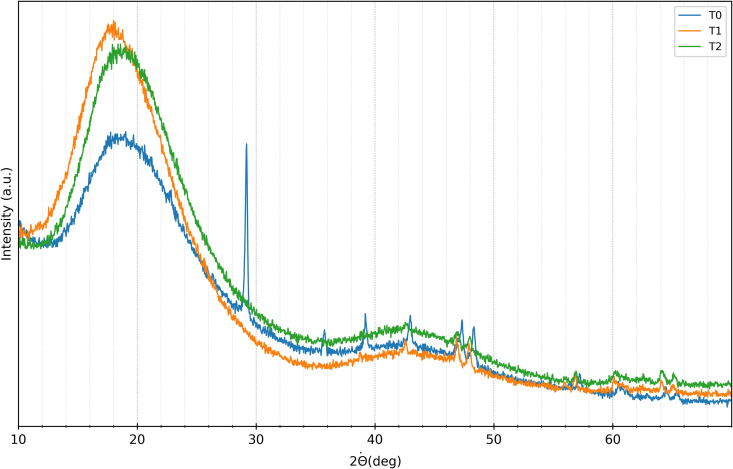
XRD SmartTrack^TM^ aligner diffractogram. Diffractrogram acquired at baseline *t*_0_ (blu), after 7 days of intraoral wear *t*_1_ (orange) and after 14 days of intraoral wear *t*_2_ (green).

**Fig 8:**
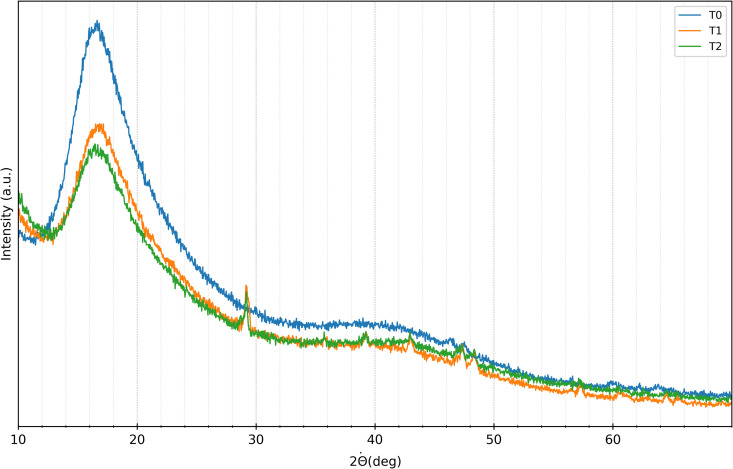
XRD Tera Harz TC-85 DAC aligner diffractogram. Diffractrogram acquired at baseline *t*_0_ (blu), after 7 days of intraoral wear *t*_1_ (orange) and after 14 days of intraoral wear *t*_2_ (green).

The results of XRD analysis performed on the aligners at baseline *t*_0_, 7 days *t*_1_, and 14 days *t*_2_, are shown in Fig 7 for SmartTrack^TM^ and in Fig 8 for Tera Harz TC-85 DAC. Visual-qualitative analysis revealed similar diffraction patterns across all time points (*t*_0_, *t*_1_, *t*_2_) for both materials, indicating a consistent amorphous structure characterized by broad peaks at 2θ angles around 16° and 40° for Tera Harz TC-85 DAC, and 18° and 43° for SmartTrack^TM^. This suggests that the fundamental crystalline structure of the aligner materials remained unchanged following intraoral exposure.

However, the diffractograms of both materials acquired at *t*_1_ and *t*_2_ exhibited sharp peaks at approximately 29°, 35°, 39°, 43°, 47°, 48°, 57°, 64°, and 65°, as shown in Fig 7 and Fig 8. These peaks, attributable to calcium carbonate (ICDD database, reference 00-005-0586), likely resulted from exposure to the oral environment, in agreement with the results of XRF analysis.

**Fig 9:**
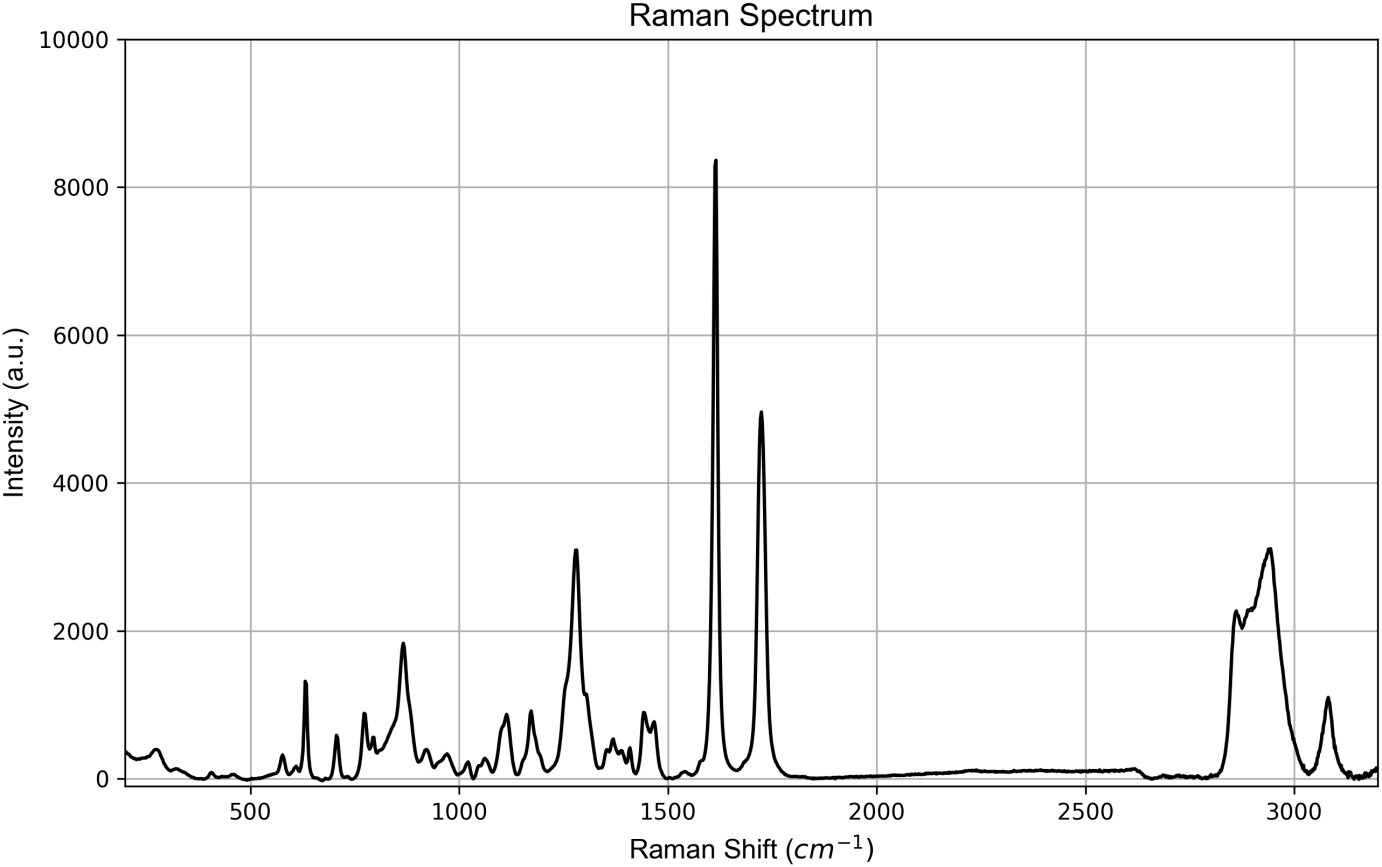
Representative Raman spectrum at *t*_0_ (baseline) of SmartTrack^TM^.

**Fig 10:**
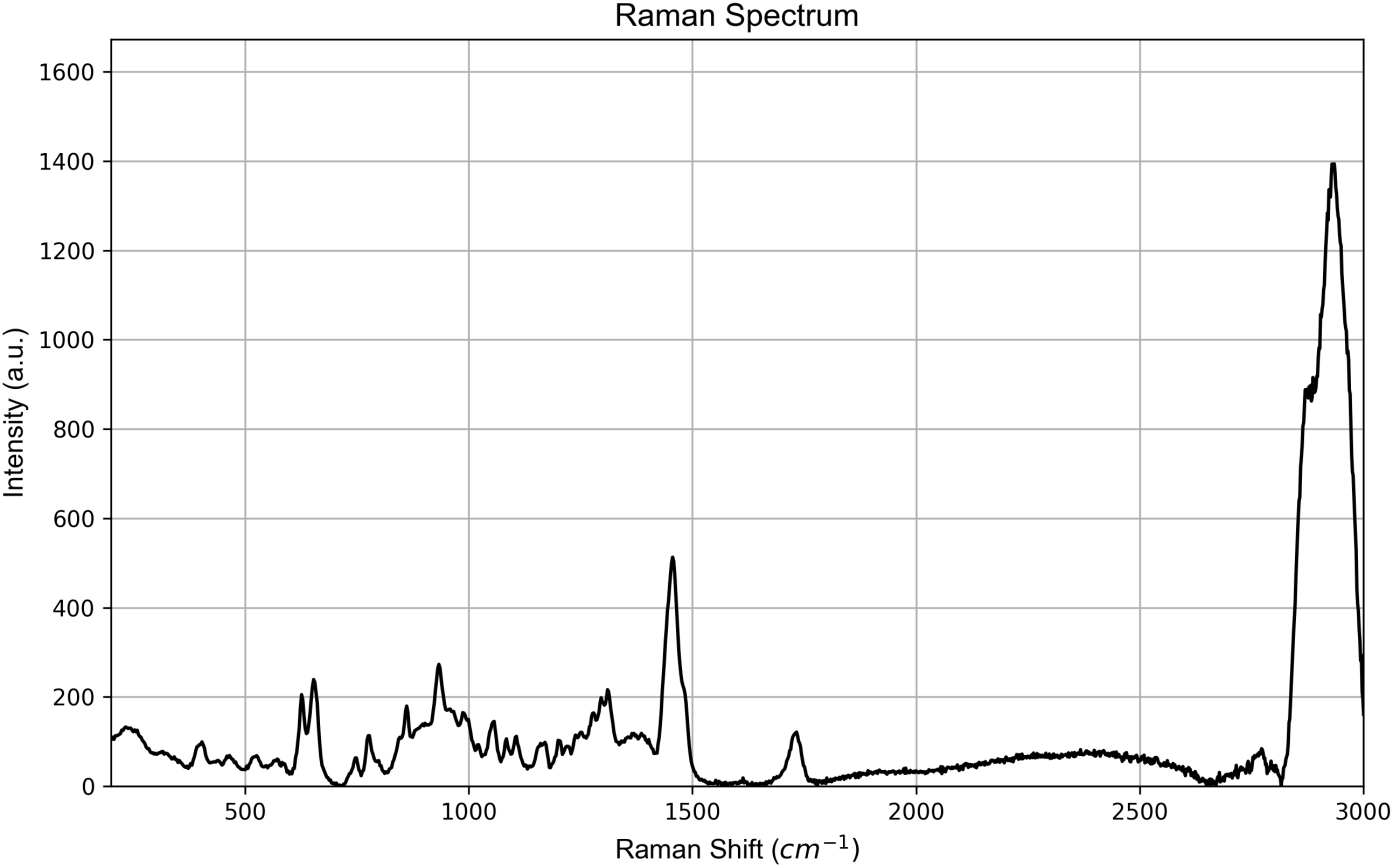
Representative Raman spectrum at *t*_0_ (baseline) of TC-85 DAC.

Raman spectra acquired at *t*_0_ on the aligners are shown in Fig 9 for SmartTrack^TM^ and in Fig 10 for Tera Harz TC-85 DAC aligner.

Key Raman bands were identified and assigned to specific vibrational modes. For the SmartTrack^TM^ spectrum, in the high wavenumber region, characteristic C-H stretching vibrations were identified at 3082 cm^-1^, 3068 cm^-1^, and 3000 cm^-1^. Additionally, a band at 2960 cm^-1^ was assigned to CH_2_ stretching. Further examination of the spectrum revealed a C=O stretching band at 1730 cm^-1^. The presence of aromatic rings was strongly indicated by a prominent C=C stretching band at 1615 cm^-1^ and C–C stretching bands at 1412 cm^-1^. Moving to the mid-wavenumber range, CH deformation was observed at 1450 cm^-1^, while CH_2_ wagging was assigned to 1370 cm^-1^. In-plane bending modes of aromatic C-H groups appeared at 1175 cm^-1^ and 1117 cm^-1^, with the latter also suggesting contributions from C–O stretching. Further evidence of C–O stretching, alongside C–C ring stretching and ring breathing modes, was evident from bands at 1282 cm^-1^, 1270 cm^-1^, 1245 cm^-1^ (shoulder), and 853 cm^-1^. A distinct band at 1038 cm^-1^ was attributed to C–C stretching of a glycol component. In the lower wavenumber region of the spectrum, significant bands included 797 cm^-1^, assigned to CH out-of-plane bending of the ring. A strong CCC in-plane bending within the ring was observed at 633 cm^-1^. Furthermore, asymmetric ring torsion was noted at 407 cm^-1^, and combined C–C stretching and CCC bending of the ring were identified at 272 cm^-1^ [35,38]. The peaks identified for SmartTrack^TM^ were consistent with those characteristic of materials based on poly(ethylene terephthalate glycol) (PETG) and polyurethane (PU) as reported in literature [39–41].

For the Tera Harz TC-85 DAC, the spectrum revealed key vibrational bands: the aromatic ring Ph-C-H in-plane bending at 626 cm^-1^, C=C stretching at 655 cm^-1^ and 746 cm^-1^, and C-C stretching (ring breathing) coupled with C-O stretching at 861 cm^-1^. Further bands included the ether group C-O-C stretching at 1106 cm^-1^, C-N stretching and N-H bending in amide III at 1279 cm^-1^, and methylene group (CH_2_) bending at 1455 cm^-1^. The urethane group *δ*(CH) and amide III appeared at 1310 cm^-1^. Ester *ν*(C=O) and urethane amide I *ν*(C=O) were observed at 1730 cm^-1^, with CH_2_ symmetric stretching at 2929 cm^-1^ and 2872 cm^-1^ [35,38,42]. The peaks identified for Tera Harz TC-85 DAC were consistent with those characteristic of polyurethane-based materials [43,44].

**Fig 11:**
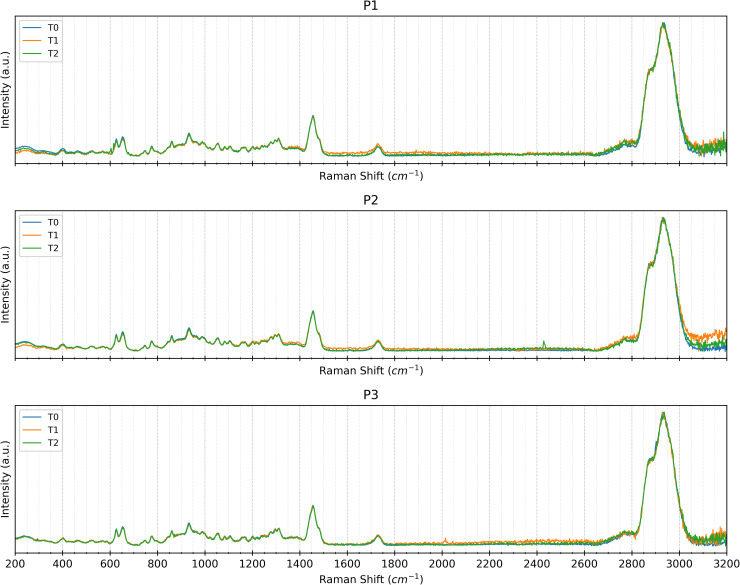
Raman Spectra at Different Time Points. Raman spectra at the three points examined (right first molar *p*_1_, left first molar *p*_2_, and incisal edge *p*_3_) of a representative Tera Harz TC-85 DAC aligner sample at baseline *t*_0_ (blu), after 7 days of intraoral wear *t*_1_ (orange) and after 14 days of intraoral wear *t*_2_ (green).

**Fig 12:**
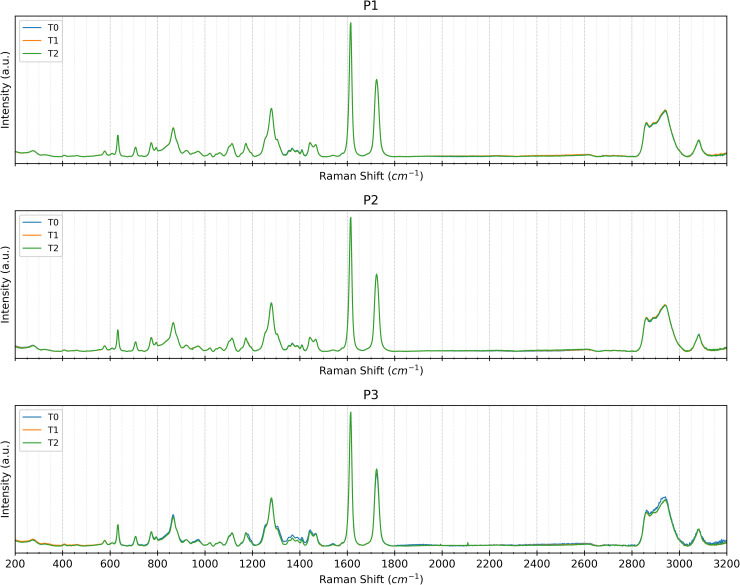
Raman Spectra at Different Time Points. Raman spectra at the three points examined (right first molar *p*_1_, left first molar *p*_2_, and incisal edge *p*_3_) of a representative SmartTrack^TM^ aligner sample at baseline *t*_0_ (blu), after 7 days of intraoral wear *t*_1_ (orange) and after 14 days of intraoral wear *t*_2_ (green).

To assess material uniformity across distinct regions, Raman spectra were acquired at *t*_0_ (before the exposure to the intraoral environment) from points *p*_1_, *p*_2_, and *p*_3_. Visual inspection of the spectra revealed no significant variations in Raman shifts across all measurement points for both SmartTrack^TM^ and Tera Harz TC-85 DAC aligners (Fig 11 and Fig 12). This absence of peak shifting, broadening, or intensity changes suggests that both materials exhibit chemical homogeneity across the examined sample and at all points of interest before intraoral exposure. Similar spectral consistency was observed after 7 (*t*_1_) and 14 (*t*_2_) days of intraoral exposure. Raman spectra acquired at (*t*_1_) and (*t*_2_) shown in Fig 11 and Fig 12 exhibited no significant variations across the investigated areas *p*_1_, *p*_2_, and *p*_3_.

**Fig 13:**
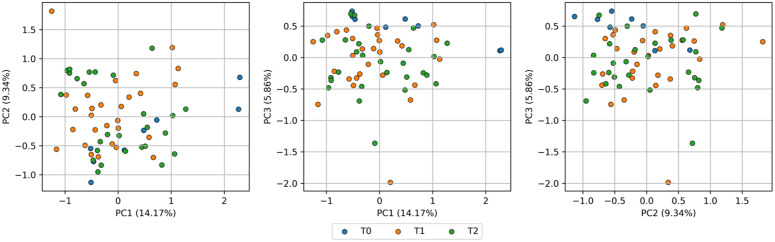
PCA results for Tera Harz TC-85 DAC aligners. Score plots of the first three principal components (PC1-PC2, PC1-PC3, and PC2-PC3) calculated from first-derivative Raman spectra in the 1500−1800 cm^-1^ region. Data points are colour-coded according to intraoral exposure time (*t*_0_, *t*_1_ and *t*_2_); the percentage of variance explained by each PC is reported in parentheses on the corresponding axes.

**Fig 14:**
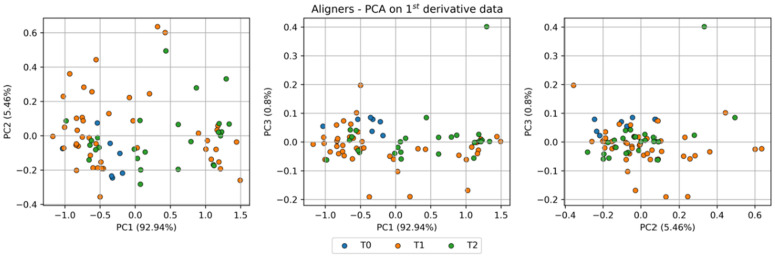
PCA results for SmartTrack^TM^ aligners. Score plots of the first three principal components (PC1-PC2, PC1-PC3 and PC2-PC3) calculated from first-derivative Raman spectra in the 1500−1800 cm^-1^ region. Measurements from (*t*_0_, *t*_1_ and *t*_2_) are colour-coded, and the percentage of variance explained by each PC is indicated in parentheses along the axes.

**Fig 15:**
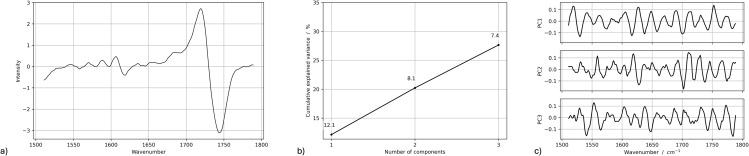
Raman PCA summary for Tera Harz TC-85 DAC aligners. **(a)** Average first-derivative Raman spectrum in the 1500−1800 cm^-1^ region. **(b)** Cumulative explained variance for the first three principal components. **(c)** Loadings of PC1-PC3 highlighting the main contributions of aromatic C=C and ester/urethane C=O bands.

**Fig 16:**
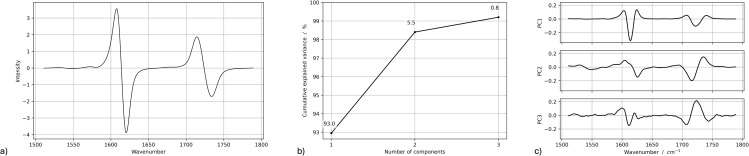
Raman PCA summary for SmartTrack^TM^ Invisalign^®^ aligners. **(a)** Average first-derivative Raman spectrum in the 1500−1800 cm^-1^ region. **(b)** Cumulative explained variance for the first three principal components. **(c)** Loadings of PC1-PC3 showing contributions from the same polyurethane-related bands and the absence of new features after intraoral exposure.

PCA was performed on pre-processed Raman spectra (first derivative, 1500–1800 cm^-1^) of both SmartTrack^TM^ and TC-85 DAC aligners to identify potential variations induced by intraoral exposure. The resulting score plots, presented in Fig 13 for the direct 3D-printed aligners and in Fig 14 for the thermoformed Invisalign aligners, display data points color-coded according to the different times (*t*_0_, *t*_1_, *t*_2_). Fig 15 and Fig 16 report, for TC-85 DAC and SmartTrack^TM^, respectively, the average first-derivative Raman spectrum in this region, the cumulative variance explained by the first three principal components, and the corresponding loading profiles, providing a quantitative summary of how spectral information is distributed among PCs and which spectral features drive the modeled variance. In particular, the cumulative variance plots show that, for Invisalign SmartTrack^TM^, the first three principal components (PCs) account for approximately 99% of the total spectral variance (PC1 ≈ 93%, PC2 ≈ 5.5%, PC3 ≈ 0.8%), whereas for the 3D-printed TC-85 DAC aligners the same components explain about 27% of the variance (PC1 ≈ 12.1%, PC2 ≈ 8.1%, PC3 ≈ 7.4%). The marked difference in cumulative variance between the two materials likely reflects intrinsic differences in spectral complexity and signal distribution. In particular, the higher variance concentration in SmartTrack^TM^ suggests that most spectral information is dominated by a limited number of highly intense bands, whereas the lower cumulative variance observed in TC-85 DAC indicates a more distributed contribution of multiple spectral features across principal components. Despite these differences in variance distribution, the score plots for both materials (PC1–PC2, PC1–PC3, PC2–PC3) show a substantial overlap of measurements collected at *t*_0_, *t*_1_ and *t*_2_, with no distinct clustering or systematic trends along any principal component. The corresponding loading plots indicate that, in SmartTrack^TM^, PC1–PC3 are dominated by the aromatic C=C stretching band around 1600 cm^-1^ and by the ester C=O stretching near 1720–1730 cm^-1^, whereas in TC-85 DAC the same principal components are mainly influenced by urethane-related C=O stretching in the 1720–1730 cm^-1^ region together with aromatic and aliphatic C–C/C–H vibrations around 1600 cm^-1^. For both aligner materials, these material-specific contributions remain stable at all time points and no additional bands appear after intraoral wear.

Taken together, the limited separation in the score space and the similarity of the loading patterns across *t*_0_, *t*_1_ and *t*_2_ support the absence of systematic changes in peak position, width, shape or relative intensity attributable to 7–14 days of intraoral exposure for either SmartTrack^TM^ or TC-85 DAC aligners. As a basic quantitative summary, the variance explained by the first three PCs and the lack of time-dependent grouping in the score plots indicate that the residual variability is mainly intra-sample noise rather than a systematic effect of intraoral ageing, consistent with the elemental (XRF) and structural (XRD) results. It should be noted that PCA is an exploratory multivariate technique aimed at identifying patterns in complex datasets and does not constitute a formal hypothesis-testing approach; therefore, the interpretation of clustering or overlap is based on descriptive evaluation of score distributions.

## Discussion

Even though thermoforming is one of the most common methods for producing clear aligners, direct 3D printing is emerging as a promising alternative in contemporary orthodontics. As this new technique entered the market, it is important to understand how 3D-printed aligners behave when exposed to the oral environment, considering their reaction to mechanical, thermal, and chemical conditions. To assess their performance, it is useful to compare them with traditional thermoformed aligners, which are currently the most widely used. Studying the chemical changes in aligners is important to understand their long-term stability and performance: poor chemical stability can weaken their mechanical properties, resulting in a reduced effectiveness of orthodontic treatment. Furthermore, changes in the chemical composition of aligners may affect their transparency and color, impacting aesthetics. Understanding the chemical stability of aligners is considered crucial also from the safety point of view. Existing papers showed that materials with low chemical stability can degrade over time, potentially releasing substances into the mouth, which may pose risks, especially for individuals with allergies [34,45]. Other factors relevant to clinical performance, such as printing parameters (layer thickness and build orientation), also appear to influence both mechanical properties and color stability of 3D-printed aligners, indicating that material behavior can substantially vary depending on the printing protocol [46].

The aim of the present study is to evaluate the intraoral aging effects on two different clear aligner materials (Invisalign® SmartTrack^TM^ and Tera Harz TC-85 DAC) employing a multi-analytical approach. Optical Microscopy, Raman spectroscopy, XRF, and XRD were used to assess changes in the chemical and structural stability of these materials after intraoral exposure over 7 and 14 days.

Optical microscopy analysis allowed us to validate the actual use of clear aligners by patients, as the working surfaces clearly show signs of wear on *t*_1_ and *t*_2_ compared to *t*_0_.

In the case of SmartTrack^TM^, microcracks are observed at *t*_1_ and *t*_2_, which are not present at *t*_0_. Additionally, the surface at *t*_0_ appears glossier compared to the later time points. For both materials, the aligners appear translucent at *t*_0_, while at *t*_1_ and especially at *t*_2_, they become increasingly opaque. This increased opacity is likely due to the accumulation of plaque and calcium deposits on the aligner surface over time.

For the TC-85 DAC aligners, the surface features observed at *t*_0_ are due to the manufacturing process. Indeed, the resin is placed semi-polymerized onto the metallic plate, and some unpolymerized resin flows onto the surface, capturing the plate’s texture during polymerization. However, at *t*_1_ and *t*_2_, wear areas appear and the surface becomes more opaque compared to the unworn regions of the aligner. This highlight that the use induced some modification to the surface morphology of the aligners.

XRF analysis showed a significant similarity in the elemental composition of SmartTrack^TM^ and Tera Harz TC-85 DAC aligners after use, indicating that exposure to the oral environment led to the localised calcification of the biofilm on the surface aligners by a possible interaction with intraoral conditions, due to mineral deposition from saliva or food. These results have been confirmed also by XRD analysis, where the presence of calcium carbonate were observed at *t*_1_ and *t*_2_. Despite these interactions, the overall amorphous structure of both aligners remained unchanged, as indicated by consistent diffraction patterns over time. This suggests that the core structural integrity of the polymers was preserved even after intraoral exposure.

Further confirmation of the chemical stability of both materials came from Raman spectroscopy. Spectral analysis across different time points (*t*_0_, *t*_1_, and *t*_2_) revealed no significant shifts, broadening, or changes in peak intensity within the three examined regions (*p*_1_, *p*_2_, and *p*_3_). This indicates that neither SmartTrack^TM^ nor Tera Harz TC-85 DAC undergoes substantial chemical degradation over the studied period. Even if the exploratory nature of PCA should be considered when interpreting these findings, PCA analysis of the Raman spectral data showed no distinct separation among the three analysed regions. It is with noticing that, even if some signs of wear have been detected on the surface by optical microscopy, the occurrence of these damage sources cannot be distinguished with Raman spectroscopy which did not detected any chemical changes in the materials.

The absence of significant structural or chemical changes in both materials reinforces their stability and suitability for clinical application. However, these findings should be interpreted considering the study’s limitations: patient-related variables were not standardized or quantitatively assessed (which may introduce inter-individual variability not captured by the present design) and the relatively short intraoral exposure period (14 days) may not fully capture long-term degradation mechanisms, such as polymer fatigue, wear, or biofilm formation. Despite this, the authors decided not to investigate periods longer than 14 days because, for SmartTrack^TM^ aligners, protocols that provide for the continuous use of a single aligner for a maximum of 14 days are currently commonly used [47]; so, also in order to be able to perform the comparison with the aligners printed directly in 3D, it was decided not to extend the study for more than 14 days.

The obtained results are concordant with data from multiple studies in the literature that have assessed the chemical aging of aligners using various techniques, including FTIR and Raman spectroscopy [45,48]. Indeed, both Invisalign® SmartTrack^TM^ and Tera Harz TC-85 DAC aligners exhibit high chemical and structural stability after 7 and 14 days of intraoral use.

In line with previous studies, our findings confirm that clear aligners are generally safe and do not exhibit measurable degradation during short-term intraoral use. Nonetheless, it is important to consider potential cumulative effects that may arise under prolonged or challenging oral conditions. Some authors have reported slight to moderate cytotoxicity, particularly when aligners are subjected to extended aging protocols or long-term immersion in saliva-like or ethanol solutions, conditions that exceed standard clinical wear schedules and can promote polymer degradation and release of trace compounds [49,50]. Although clear aligners typically facilitate better oral hygiene compared to fixed appliances, and proper fitting minimizes gingival irritation, their outcomes still heavily depend on patient compliance. Inadequate cleaning can lead to biofilm accumulation, increase microbial load, and potentially accelerate surface deterioration. Therefore, appropriate home-care protocols are crucial to limit bacterial colonization and prevent surface calcification [51,52]. Mechanical stability may also decline with extended exposure. Both in vivo and simulated aging studies report reductions in elastic modulus, tensile strength, and force delivery due to cumulative fatigue, viscoelastic relaxation, and repeated masticatory loading. Longer exposure can further promote microcrack formation and material softening [33,53]. These effects are among the reasons that justify the standard clinical practice of replacing aligners every 1–2 weeks to maintain effective biomechanics.

In this context, studies in existing literature about the evaluation of the chemical stability of aligners made by direct 3D printing are, to date, rather scarce compared to those dedicated to thermoformed aligners. Further investigations are needed to be undertaken in this area. These findings highlight the importance of continued research into the chemical stability of orthodontic aligners, particularly in the realm of 3D-printed aligners, where data remains limited. Ensuring that aligners maintain their chemical integrity over time is essential for preserving their mechanical properties, aesthetic appeal, and biocompatibility, ultimately leading to improved patient outcomes. Future studies should further investigate long-term chemical and mechanical stability, particularly for newer 3D printed aligner materials, to optimize their clinical performance and ensure patient safety, also in view of their possible use as a removable retainer.

## Conclusion

This study confirms the high chemical and structural stability of both thermoformed (Invisalign® SmartTrack^TM^) and 3D-printed (Tera Harz TC-85 DAC) aligners after 7 and 14 days of intraoral use. No significant chemical degradation or structural changes were observed, supporting their reliability for clinical application.

XRF analysis showed a similar elemental composition in both aligners, and XRD analysis confirmed the amorphous structure of both aligners, demonstrating that the structural integrity remained unchanged over time. Calcium carbonate was detected after use, likely due to intraoral interactions.

Raman spectroscopy analyses showed, for each patient and for both types of aligners, a very high similarity of spectra at all timepoints and in all positions examined.

Therefore, within the limits of this study, intraoral exposure for up to 14 days did not result in detectable changes in the surface chemical composition of the aligner materials. However, patient-related factors such as diet, saliva composition, and compliance were not directly controlled and may influence material behavior under different clinical conditions.
